# Analysis of capivasertib via ion-pairing with erythrocin B as a spectrofluorometric probe

**DOI:** 10.1038/s41598-026-49688-5

**Published:** 2026-05-01

**Authors:** Hesham Salem, Hytham Raafat, Alyaa Alaa, Belal M. Abdelghany, Anas Mahmoud, Abdelrahman Medhat, Omar Saied, Michael Amir, Amany Abdelaziz

**Affiliations:** Pharmaceutical chemistry department, faculty of pharmacy, Deraya University, New Minia, Egypt

**Keywords:** Capivasertib, Resonance Rayleigh scattering, Erythrosin B, Green analytical chemistry, Eco-Scale, NEMI, GAPI, AGREE, AGREE-prep, RGB12, BAGI., Chemistry, Environmental sciences

## Abstract

A novel, eco-friendly spectrofluorimetric method has been developed for the sensitive determination of capivasertib, based on resonance Rayleigh scattering enhancement. Unlike previously reported methods, this approach utilizes erythrosine B as a safer ion-pairing reagent in an acidic aqueous medium, eliminating the need for organic solvents or complex extraction steps. The interaction between capivasertib and erythrosine B leads to a stable ion-pair complex, producing a strong Rayleigh scattering signal measurable at λex530/λem550 nm. The method demonstrated excellent linearity over the range of 20–2000 ng mL⁻¹, with a detection limit of 5.61 ng mL⁻¹ and a quantification limit of 17 ng mL⁻¹. All analytical parameters were optimized and validated according to ICH guidelines. To holistically substantiate sustainability, the method’s environmental and practical profiles were appraised using all contemporary greenness/whiteness/applicability metrics employed in this work: Analytical Eco-Scale (ESA), NEMI pictogram, GAPI, AGREE, and AGREE-prep for sample-preparation greenness; the White Analytical Chemistry RGB12 model (whiteness); and the Blue Applicability Grade Index (BAGI) (blueness/applicability). This work introduces a greener, simpler, and more accessible alternative for capivasertib analysis, with potential applications in pharmaceutical quality control and routine laboratory testing.

## Introduction

**Fig. 1:**
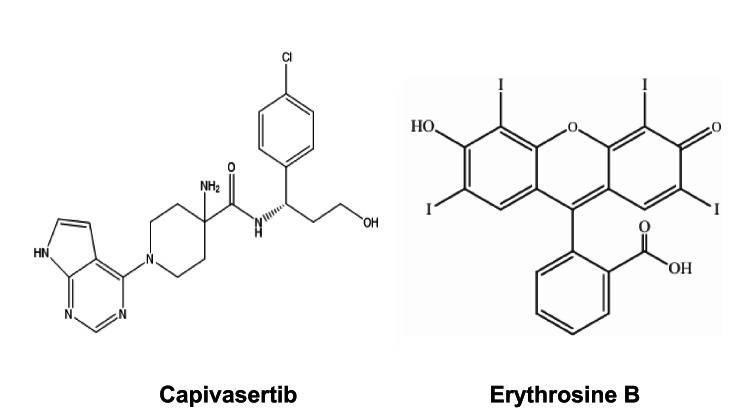
Molecular structures of capivasertib and Erythrosine B dye

In recent years, green analytical chemistry has gained significant attention due to its emphasis on environmentally sustainable materials, chemicals, and procedures^1–4^. This shift has led to the development of tools that quantitatively and qualitatively assess adherence to green and sustainable principles^5–7^. Among these tools are the Eco-Scale Assessment (ESA)^8^, National Environmental Methods Index (NEMI)^9^, Green Analytical Procedure Index (GAPI)^10^, and Analytical Greenness Calculator (AGREE)^11^. The AGREE-prep tool^12–14^ was also employed to evaluate the environmental responsibility of sample preparation. Erythrosin B (Fig. 1), a xanthene-based dye, is widely used in pharmaceutical and biomedical applications due to its strong protein binding and natural emission properties. It has been applied in the analysis of proteins^15,16^, vitamins^17^, and various therapeutic agents^18–23^. Ion-pair complexation with nitrogenous compounds has been a common strategy in these applications, particularly using dyes like eosin Y and erythrosin B^24–32^. Capivasertib (CAP, Fig. 1) is a potent, selective, orally bioavailable pan-AKT kinase inhibitor that has recently attracted considerable clinical interest. Pharmacologically, CAP exerts its therapeutic effect by inhibiting all three isoforms of AKT (AKT1, AKT2, AKT3), a central node in the PI3K/AKT/mTOR signaling pathway, which is frequently deregulated in various malignancies^33^. By blocking AKT-mediated phosphorylation cascades, CAP suppresses key cellular processes such as proliferation, glucose metabolism, and survival pathways, ultimately promoting apoptosis in cancer cells^33,34^. Its clinical relevance has been further highlighted by ongoing investigations and approvals for its use in treating certain forms of breast cancer, particularly in combination with endocrine therapy. Despite the growing therapeutic importance of CAP, only two analytical methods have been published for its determination: a spectrofluorimetric method^27^ and an LC–MS method^35^. Both approaches, however, involve drawbacks such as the use of hazardous solvents, complicated sample preparation, or limited accessibility in routine laboratories. Therefore, there remains a need for a sensitive, simple, and environmentally benign analytical method for CAP quantification. The present study aims to develop a novel, sustainable spectrofluorimetric method for the determination of capivasertib based on ion-pair complex formation with erythrosin B. The proposed method offers high sensitivity, excellent environmental compatibility, and compliance with ICH validation guidelines^36^. Furthermore, it avoids hazardous reagents and demonstrates successful applicability to pharmaceutical formulations, making it a strong candidate for routine quality-control analysis. Also, RRS study is the elastic scattering of light or electromagnetic radiation by particles (molecules, atoms) much smaller than the radiation’s wavelength, such as air molecules. It causes shorter blue/violet wavelengths to scatter more intensely.

## Experimental

### Instruments

The spectrofluorimetric measurements were carried out using a JASCO FP-83 Spectrofluorimeter. The device features a 400 V PMT and a 150 W Xe-arc light. The excitation and emission monochromators featured a 5 nm slit width via a rate of scanning 1000 nm/min. All measurements were carried out utilizing quartz cells with 1 centimeter diameter. The Aquatron 4000d water still (Cole-Parmer, Stafordshire, UK) was utilized for production of double-distilled water.

### Reagents and solvents

Erythrosine-B was purchased from MP Biomedicals LLC; 99.12% purity (Illkirch, France) and prepared by dissolving 50 mg in 250 mL of double-distilled water to give a concentration of 2.27 × 10− 4 M. Erythrosine B reagent should be stored in a cool, dry, well-ventilated area, preferably in its original, tightly sealed container. Avoid direct sunlight and protect from physical damage. If a solution is prepared, it is recommended to store it no longer than one day. CAP (purity ≥ 98%) were purchased from Med Chem Express. TRUQAP capivasertib 200 mg film coated tablet blister pack (407960).

### Stock and Working solutions

Solvents such as acetone, ethyl alcohol, DMF, methanol, and acetonitrile were provided by El Nasr Co., Cairo, Egypt. An acetate buffer solution was prepared by mixing appropriate quantities of acetic acid (0.1 M) and sodium acetate (0.1 M), resulting in buffer solutions with a pH range of 3.5–4.5. To achieve the desired pH, these components were combined in various ratios while maintaining a total buffer strength of 0.1 M. All solvents, compounds, and chemicals used were of analytical grade and were not further purified. Fresh solutions were prepared daily.

### Procedure

To prepare the stock solution of CAP, 10 mg of CAP was weighed and placed inside a volumetric flask of 100 mL, combined via the requisite volume of distilled water, and subsequently filled to the mark with more distilled water, resulting in a solution of 100 µg mL^− 1^. Consequently, the specified quantity of the aforementioned stock was serially diluted with distilled water to generate a working solution. The arranged solutions were kept refrigerated (2–8 °C) only for short interim periods during analytical runs to avoid any possible degradation.

### Steps involved in the system

The experimental procedures based on the RRS approach were carried out using aliquots of CAP in 10.0 mL volumetric flasks, covering a concentration range of 20–2000 ng mL⁻¹. Each tube was supplemented with 1.0 mL of acetate buffer and 1.2 mL of erythrosine B solution (227.3 µM), followed by the addition of distilled water. The mixture was gently agitated and allowed to stand for 4 min. A certified reference standard of CAP (purity ≥ 98%) was obtained from Med Chem Express (USA) and used throughout the analysis. All procedures applied to the drug samples were similarly performed on the reference standard. A calibration curve was constructed by plotting signal intensity against CAP concentration at 550 nm, and sample concentrations were subsequently calculated.

### Application to available prescribed forms (tablets)

An amount equivalent to 10 milligrams of finely powdered Truqap^®^ tablets was transferred into a 250 milliliter calibrated volumetric flask. 50 mL of distilled water was used for sonication for 20 min. After discarding the top layer of the filtrate, water was combined to bring the solution up to par. The solvent dosages were 500, 1000, and 1500 ng mL^− 1^. This application on tablet dosage form appeared no effect of additives and excipients added during tablets manufacturing on the suggested spectrofluorometric method.

### Complex stochiometry

Reaction stoichiometry was determined using Job’s approach^37^ and the limiting logarithmic method. Two solutions of CAP and EB having the same molarity (1 × 10^− 4^ M) were prepared. The general procedure for the spectrofluorometric method was applied. The value of ΔF were plotted against the mole fraction of CAP.

## Results and discussion

### The designed system spectrum

**Fig. 2:**
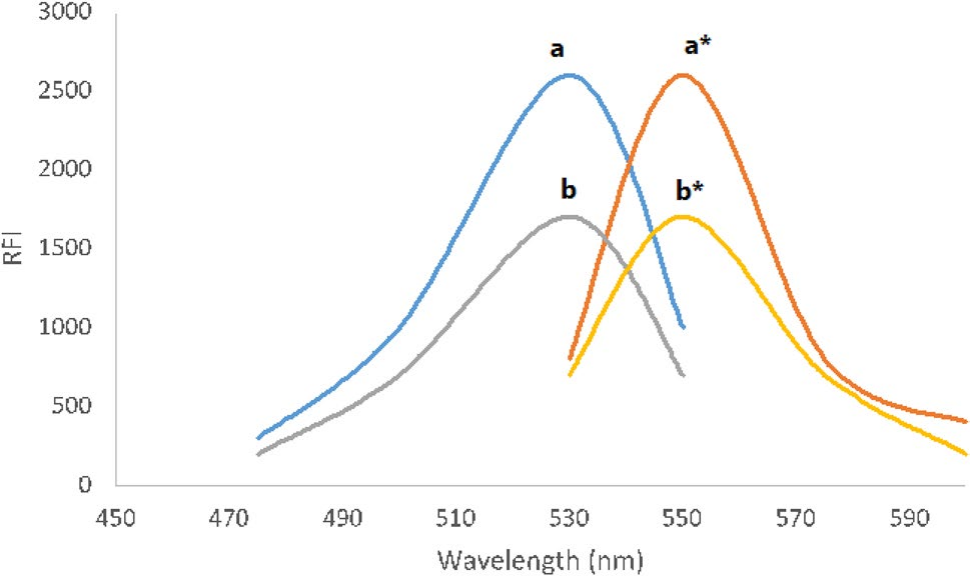
Molecular structures of capivasertib and Erythrosine B dye(a, a*) The excitation and emission spectra, respectively, of (1 × 10−4 M) erythrosine B (EB). (b, b*) The excitation and emission spectra, respectively, of the reaction product of (1 × 10− 4 M) EB and CAP (400 ng mL-1).

Forming association complexes with specific basic chemicals may reduce the maximal emission of xanthene-structured indicators (erythrosine B and eosin). The RRS signals of these dyes could be greatly enhanced by complexation with basic compounds. The alkaline center of CAP was utilized in this spectroscopic experiment to have a linked complex via the dye’s acidic center under mildly acidic conditions. Figure 2 displayed the coupled product’s and the erythrosine B dye’s RRS spectra. This figure demonstrated that the dye’s signal strength was comparatively low. The RRS spectrum emission at 550 nm showed the highest intensity and the most linear connection, indicating that an increase in CAP dose led to an decrease (Quenching effect) in RRS spectrum intensity. Because of this, the RRS approach can objectively find CAP at this wavelength.

**Fig. 3:**
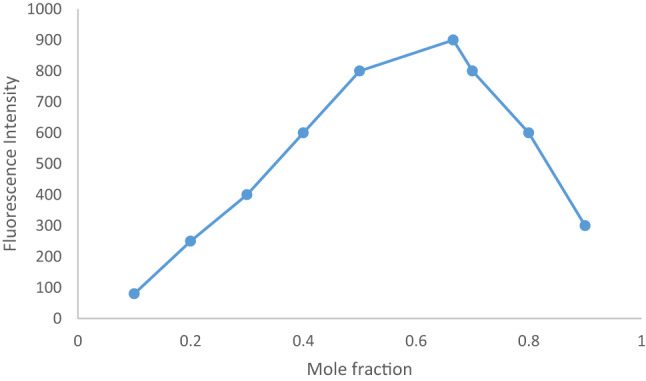
Molecular structures of capivasertib and Erythrosine B dyeJob’s plot of the ion pair complex of CAP and erythrosine using the same concentration of the analyte and the reagent (1 x10 -4 M)

### Stoichiometry of the complex (The included reactants ‘ratio)

**Scheme 1:**
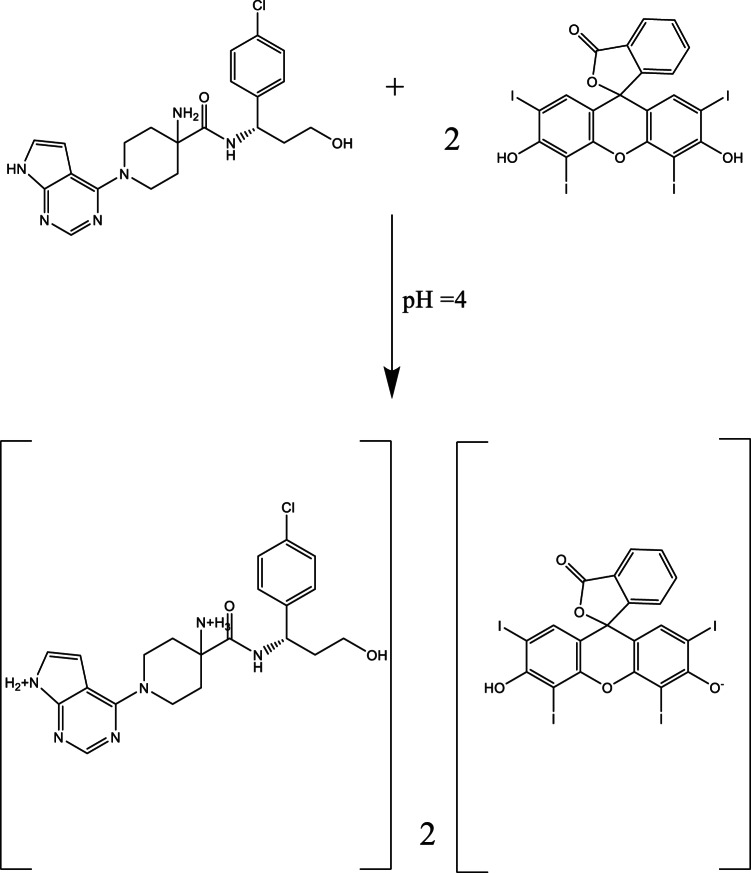
Molecular structures of capivasertib and Erythrosine B dyeThe suggested mechanism for the binary complex formation between CAP and EB.

**Fig. 4:**
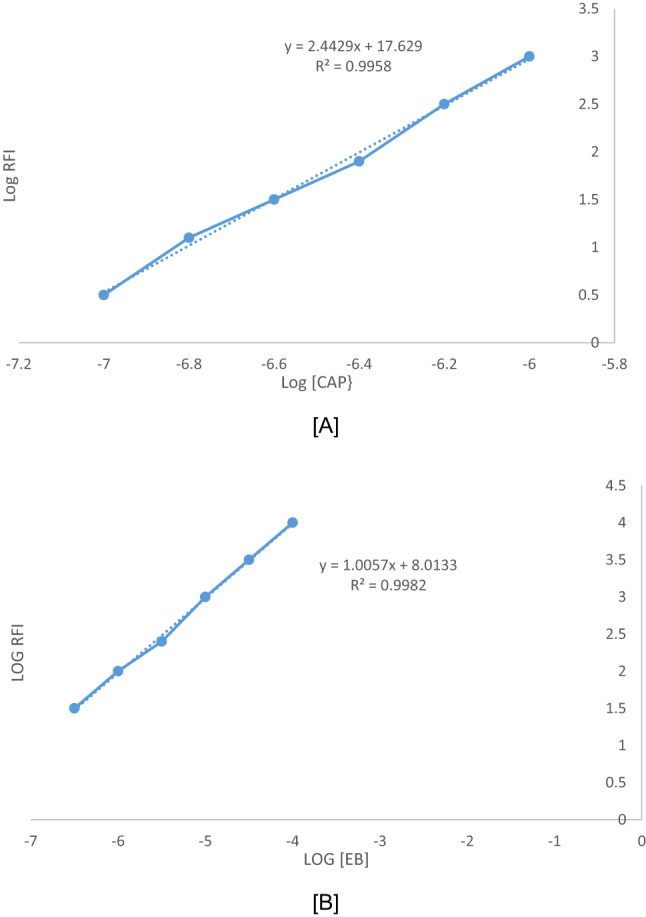
Molecular structures of capivasertib and Erythrosine B dyeStoichiometry of the fluorometric interaction between CAP and EB using logarithmic method where [**A**] log CAP against log RFI, [**B**] log EB against log RFI

The stoichiometry of the CAP–EB complex was investigated using Job’s method and the limiting logarithmic method, revealing a 2:1 molar ratio (CAP: EB), *(*Scheme 1*)*. This stoichiometry is consistent with the ionization behavior of the functional groups involved, which is governed by their respective pKa values. EB contains two acidic groups, phenolic and carboxylic. The phenolic group has a lower pKa due to its position between two electron-withdrawing iodine atoms, making it more acidic and thus more readily ionized in mildly acidic conditions (pH 3.5–4.5). This leads to the formation of a monovalent anion (EB⁻), which is available for electrostatic interaction. CAP contains two amino groups that can be protonated in acidic media, forming a dicationic species (CAP²⁺). The pKa values of these amino groups are sufficiently high to ensure their protonation under the experimental conditions. Therefore, in acidic aqueous solution: EB exists predominantly as a negatively charged anion due to phenolic ionization. CAP exists as a positively charged cation due to amino group protonation. This electrostatic attraction leads to the formation of a stable ion-pair complex, with a stoichiometry of 2 CAP molecules per 1 EB molecule, consistent with the observed slope values from the limiting logarithmic method (2.44 for CAP and 1.00 for EB), *(*Fig. 4*)*.

### Optimization reaction factors

By examining and adjusting the response items that touched the signal amplitude of the RRS, the spectroscopic measurements were optimized.

#### The effects of pH and buffer volume

**Fig. 5:**
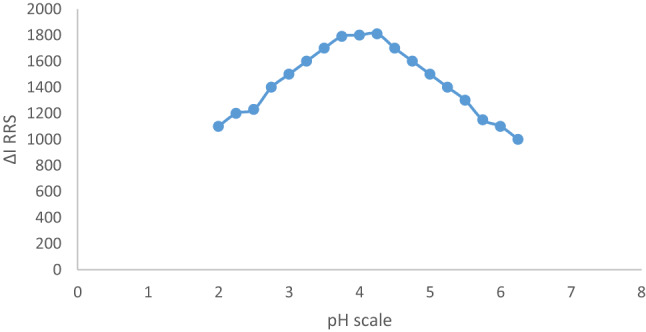
Molecular structures of capivasertib and Erythrosine B dyeThe impacts of pH on the complex’s signal amplitude.

**Fig. 6:**
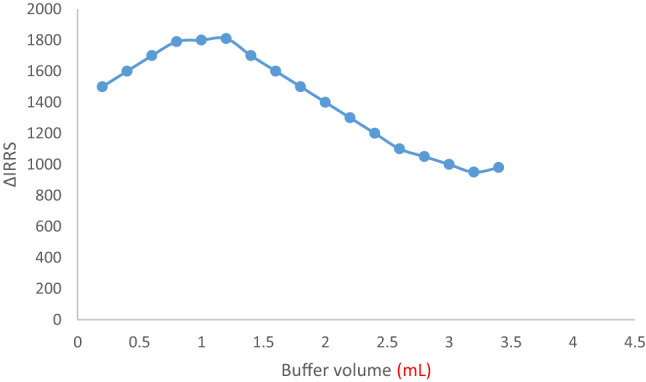
Molecular structures of capivasertib and Erythrosine B dyeThe impacts of buffer quantity on the complex’s signal amplitude.

The materials were tested at pH values between 2.0 and 6.5 in order to examine the formation of a CAP-erythrosine B complex. The pH of the sample significantly affects the reaction of the CAP-erythrosine B complex. The highest *Resonance Rayleigh Scattering (RRS)* amplitudes in the system under study were found in the pH range of 3.5 to 4.5. When the pH was changed, the response values significantly increased as viewed in Fig. 5. The proper pH was found to be 4.0 and the buffer volume had a great effect on the forming of the binary complex. The response of the binary complex was examined utilizing the acetate buffer from 0.25 to 3.5 mL volumes. Using an acetate buffer system at levels ranging from 0.25 to 3.5 mL, the binary complex response was investigated. Controlling buffer levels among 0.8 and 1.2 mL produced the strongest reaction (greater emission). When there were significant volume changes, the response levels decreased. Since the large volume of the buffer allow the pigment’s negative charge to oppose along the positively charged component of the buffer for coupling, delaying the complex initiation, a suitable buffer volume was required to keep a consistent pH level. Because of these characteristics, 1.0 mL was the ideal amount for this scenario (Fig. 6).

#### Impacts of the erythrosine B stain volume and reaction incubation time

**Fig. 7:**
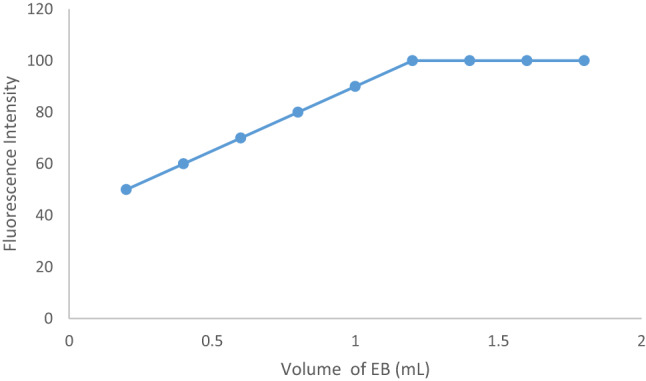
Molecular structures of capivasertib and Erythrosine B dyeEffect of EB volume (2.724 x10-5 M) on the fluorescence intensity of CAP-EB complex.

**Fig. 8:**
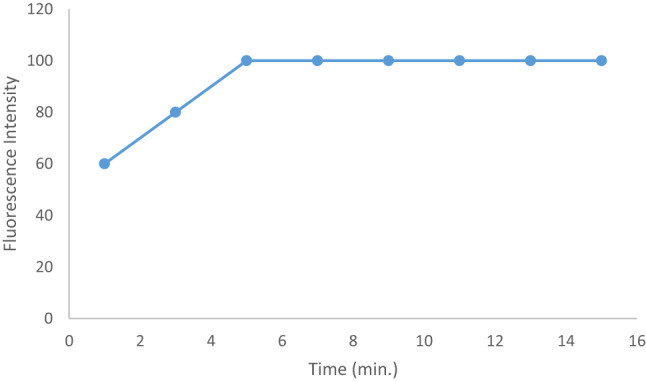
Molecular structures of capivasertib and Erythrosine B dyeEffect of time on the fluorescence intensity of CAP-EB complex.

When employing this method, it was crucial to experiment with different concentrations of the CPB stain in order to obtain the strongest result. The optimal concentration of EB was found to be 2.724 × 10^− 5^ M, which corresponds to 1.2 mL of the working solution (Fig. 7). The stain solution with the highest reaction record was the one that contained 1.2 milliliters. Because of the low dye concentrations, the partial reaction produced a poor response. Increasing the concentration has a declining impact since the dye has a tendency to self-agglomerate. The CAP–EB complex was found to form rapidly at room temperature, and based on preliminary kinetic observations, the RRS signal stabilized within 5 min of mixing. Therefore, all measurements were recorded after a 5-minute incubation period, which was deemed sufficient for complete complex formation and consistent signal acquisition (Fig. 8).

#### The dispersing liquid impact

**Fig. 9:**
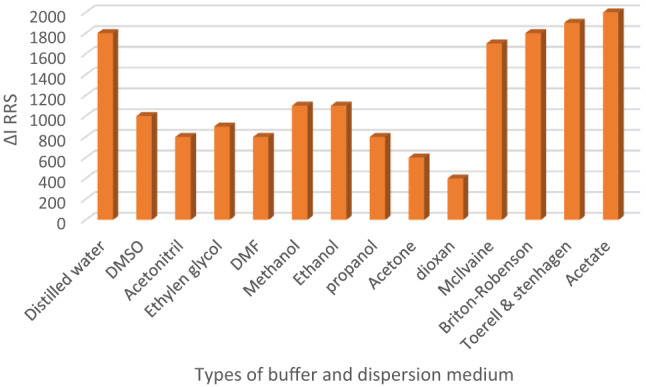
Molecular structures of capivasertib and Erythrosine B dyeThe regulating buffer type and dispersing liquid influence on the RRS signal magnitude of the ion-coupling complex developed between CAP and Erythrosine B.

Distilled water, dioxan, and alcohols (methanol, ethan-2-ol, and propane-2-ol) were among the dispersing solvents whose efficacy was examined (Fig. 9). When distilled water was used as the last diluting solvent in the procedure, the RRS signal enhancement rates peaked. The organic medium’s low measurement scores added to the solvents’ detrimental effects on the final system. Certain solvents have the potential to change the RRS signal, which could disrupt the proposed technique. Ethan-2-ol and methyl alcohol, for instance, are short-chain solvents that dissolve and interfere with system growth in watery conditions^38^. High concentrations of alcohols, such as methanol and ethanol, were found to interfere with the complexation process, likely due to their impact on solvent polarity and solute solubility. This interference can hinder complex formation and reduce system stability. It was helpful that water was discovered to be the most effective diluent, even though this was regrettable in terms of how environmentally friendly the process was. Compared to the other solvents, water has a greater polarity and dielectric constant (80.2 and 9.0, respectively). The majority of the system’s components were soluble in water, indicating that they were quite miscible with one another. The synthesis of complex components in the system may be hindered by a low miscibility among various organic solvents with differing dielectric constaFnts. Although the study promotes green chemistry, organic solvents were briefly evaluated to confirm that water is not only the most environmentally friendly but also the most effective dispersing medium. The poor performance of organic solvents further validated water as the optimal choice.

#### Selection of the pH’s controlling solution

To determine the optimal pH for the complexation reaction, various buffer systems were evaluated at a fixed volume of 1.0 mL. The tested buffers included acetate (2.0-6.5), Britton–Robinson, McIlvaine, and Toerell–Stenhagen, (3.0–6.0) for all. Among these, the acetate buffer at pH 4.0 provided the highest RRS signal intensity and the most stable complex formation, indicating its superior compatibility with the system. Therefore, acetate buffer at pH 4.0 was selected as the optimal pH-controlling solution (Fig. 9).

### Validation of the method

The International Council for Harmonization’s (ICH) requirements were assessed for conformance with the current RRS-based approach^36^. Several statistical properties, including accuracy, linearity, precision, and resilience, were examined as part of the validation process.

#### Linearity and sensitivity

**Fig. 10:**
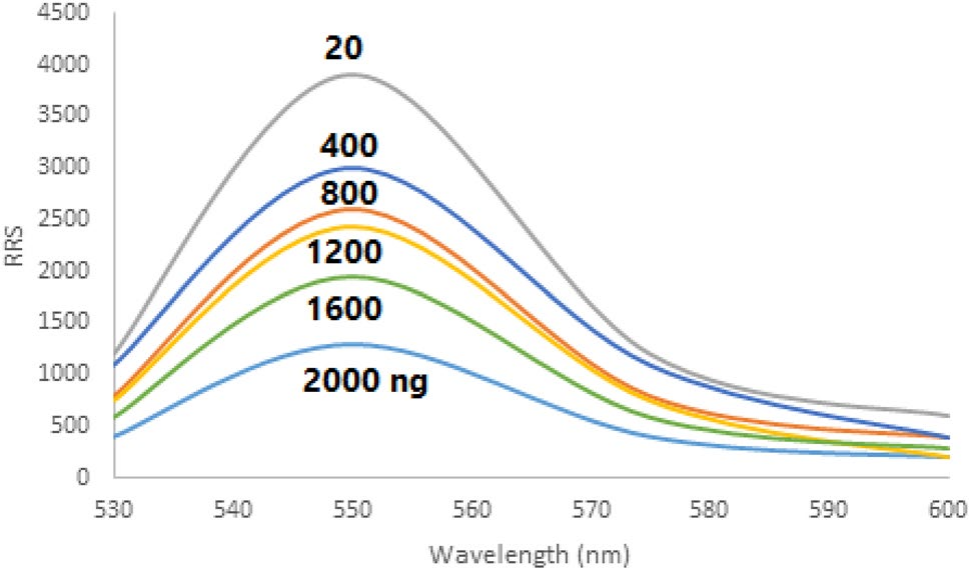
Molecular structures of capivasertib and Erythrosine B dyeAdding increased concentrations of CAP showed a quantitative decrease in the fluorescence intensity of EB.

Adding increased concentrations of CAP showed a quantitative decrease in the fluorescence intensity of EB (Fig. 10).

To investigate the CAP samples’ RRS reaction upon complexation with the dye, a spectroscopic examination was carried out. A calibration plot was produced by comparing the Δ RRS data to the CAP dose. Between 20 and 2000 ng mL^− 1^, the suggested approach’s response was linear. Table 1 illustrates the outcomes of a linear regression analysis conducted with the aforementioned technique.

**Fig. 11:**
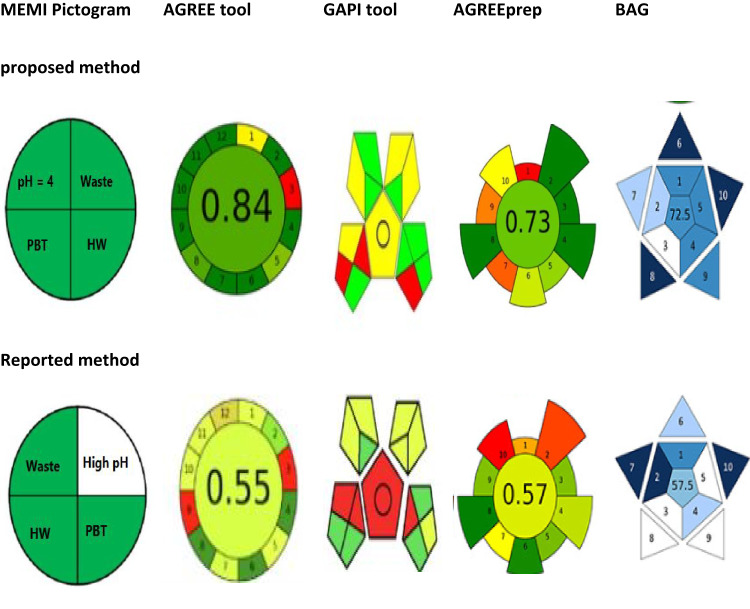
Molecular structures of capivasertib and Erythrosine B dyeThe modern ecological sustainability metrics-based assessment of the current system greenness degree.

**Table 1:** The proposed resonance Rayleigh scattering technique analytical data of CAP.

Parameter	Value
Linearity rang (ng mL^− 1^)	20–2000
Slope	3.20
Intercept	70.75
Determination coefficient (r^2^)	0.9997
Number of determinations	6
Standard deviation of residuals (S_y/x_)	23.98
Limit of quantitation (ng mL^− 1^)	17.00
Limit of detection (ng mL^− 1^)	5.61

##### Detection and quantitation limits

Utilizing the proper formulas, the technique’s sensitivity levels—including the quantification and detection limits—were determined. LOQ=10SD/S and LOD = 3.3SD/S Here, SD implies the intercept’ standard deviation, and S implies the slope. The LOQ and LOD outputs were 17.00 ng mL^− 1^ and 5.61 ng mL^− 1^, subsequently, after the data analysis was completed (Table 1).

#### Precision and accuracy

Utilizing three doses (100, 1000, and 1500 ng mL 1), the precision of the RRS-based approach was assessed. The standard deviation and the proportion of retrieved samples are often used metrics to assess accuracy. Table 2 shows that the method produces extremely precise results. The inter-day and intra-day precisions of the current method at 100, 1000, and 1500 mg mL^− 1^ were also examined using the suggested technique. The precision of the procedure was evaluated using the %RSD values. Table 2 shows that the current method has high precision with RSD of less than 2%.

**Table 2:** Accuracy and precision assessment of the planned Rayleigh scattering technique for analysis of CAP.

Parameter	ng mL^− 1^	% Recovery* ± RSD
**Accuracy**	100	100.68 ± 0.89
	1000	99.50 ± 1.37
	1500	101.35 ± 1.73
**Inter-day precision**	100	98.47 ± 0.96
	1000	100.49 ± 1.48
	1500	99.49 ± 0.99
**Intra-day precision**	100	99.04 ± 1.11
	1000	100.97 ± 0.85
	1500	99.99 ± 1.04

*Mean of three determinations, RSD: Relative standard deviation.

#### The system’s robustness testing

To determine if the intended RRS approach could result in fluctuations in the tracked RRS signal strength, the response, the amount of reagent, and the pH of the system were examined. The robustness of the method was assessed using these standards. The resilience of the suggested approach with respect to such intended slight adjustments was demonstrated by the results of RRS augmentation measurements, which were sincere via minor changes (Table 3). Consequently, the RRS measurement findings were unaffected by these minor adjustments. This demonstrated that the suggested approach was strong enough to manage the minor adjustments that were probably going to happen. The results proved the high robustness of the methods as they gave excellent %recoveries with low% RSD values.

**Table 3:** Robustness testing of the current RRS signal-based system.

Reaction variable	Reaction variable	±Value	%Rec	±SD^a^	RSD%^b^
pH (4 ± 0.1)	pH	0.1	99.42	1.66	1.67
		0.2	99.02	0.39	0.39
Buffer volume, mL (1 ± 0.1)	Buffer volume (mL)	0.9	100.87	0.96	0.95
		1.1	98.96	1.11	1.12
Dye volume, mL (1.2 ± 0.1)	Dye volume (mL)	1.1	100.39	0.63	0.63
		1.3	100.49	0.96	0.95
Time, min. (4 ± 0.5)	Time (min.)	3.5	99.18	1.06	1.07
		4.5	99.00	1.47	1.48

^a^ Mean of three determinations, SD, standard deviation.

^b^ RSD relative standard deviation.

#### Selectivity and interference

The response of the suggested approach was examined by examining the impacts of various pharmaceutical additions in order to verify whether it is selective or not. When the formulation additions were present, the intended system was used to analyze the CAP analytes. This had no appreciable influence on the outcomes of the approach. The interference study demonstrated that common tablet excipients did not significantly affect the RRS response. To further support this, a recovery assessment in the presence of typical formulation additives was performed, showing excellent recoveries close to 100%, confirming that these substances do not interfere with ion-pair formation or measurement of CAP. These findings verify the selectivity of the method and its suitability for routine analysis.

#### Evaluation of available prescribed formulations

The developed method and the comparison methods; spectrofluorometric^27^ and chromatographic^35^ were employed to assay CAP in tablets dosage forms. To make sure the described approach was consistent with the created one, the tests (t and F) were investigated. The precision and accuracy of the suggested fluorimetric approach and the one previously utilized to obtain the t- and F-values (95% confidence intervals) did not appear to differ (Table 4). The present RRS method outperformed earlier approaches in every way, including sensitivity, ease of use, efficiency, use of “green” liquids, and overall detection strengths. Quality controlFNE laboratories could employ the described technique to test dosage forms containing CAP because of the high recovery percentages and the absence of interference from produced package ingredients.

**Table 4:** Evaluation of trade products of the examined drug using the planned RRS method.

Dosage Form	Proposed method	% Recovery* ±SD	
		Reported method ^27^	Reported method ^35^
Truqap^®^ tablets	100.89 ± 2.02	100.95 ± 1.24	99.68 ± 1.57
t- value		0.055	0.987
F-value		2.654	1.655

*Average of 6 determinations. Tabulated values at 95% confidence limit are t = 2.306, F = 4.76.

### System greenness assessment

A popular topic in scientific society right now is the effects of chemical processes on environmental and human durability. As a result, while developing a diagnostic technique to measure a certain component, it is crucial to take into account both the environmental impact of the system and the metrological value of the data^39–55^. It is crucial to note that a variety of approaches have been selected as practical measures to evaluate environmental viability.

#### Analytical eco-scale score application

The analytical ESA is a numerical scale that thinks about issues including operator contact, waste disposal techniques, energy use, and reagent and waste loads. Each reagent received a penalty score determined by adding up all of the possible pictograms and warning influences. Relying on the amount of trash produced and the kind of waste treatment used, penalty points could be awarded^56,57^. The approach rating was higher (95) because the current RRS-based strategy is advantageous. This outcome made it evident that the current approach was more ecologically friendly than previously disclosed techniques.

#### NEMI pictogram employment

For the assessments, the NEMI uses a pictogram that is separated into 4 parts. These sectors covered the following topics: pH (only 2–12 permitted), residues quantities (top limit 50 mL or gm), persistent bio-accumulative harmful substances (PBT), and dangerous waste reagents. The matching region of the pictogram’s color would be green if the criterion was met. The suggested method satisfied all four of the NEMI approach’s requirements for an environmentally friendly analysis (Fig. 11). Thus, the understanding among the 2 criteria above validated the present system’s high level of environmental friendliness. The suggested method’s viability and safety were improved by using water instead of other diluents.

#### GAPI tool application

Additionally, contemporary ecological measures like GAPI and AGREE tools were utilized to evaluate the suggested approach. By combining the advantages of NEMI and ESA metrics, the GAPI methodology offers a thorough evaluation of the environmental viability of certain analytical phases in addition to a succinct description of the findings^58,59^.

#### AGREE and AGREEprep tools application

AGREE-prep, a greenness metric tool for sample preparation, uses 10 criteria to assess the environmental impact of different methods. These criteria include the use of solvents and reagents, waste generation, energy consumption, sample size, throughput, in situ sample preparation, and the use of sustainable materials. After examination, the color of each segment changes, making it simpler to see the advantages and disadvantages of each phase and how they impact the final result^12^. Insufficient greenness was achieved by the reported method for creating CAP samples. An improved method of sample preparation increased its score from 0.57 to 0.73. This improves the environment by eliminating energy-intensive heating steps, reducing solvent and reagent volumes, relying solely on water as a safe extraction medium, and shortening sonication time to minimize waste and improve operator safety.

### Whiteness evaluation (RGB12 tool)

The RGB12 design^60–62^, that integrates 3 different color groups, is used to evaluate the whiteness of the analytical technique. Blue denotes the success of economic advancement, green denotes commitment to green chemical basis, and red denotes analytical efficiency. When evaluated using a green evaluation, the new fluorescence analysis method shows a better degree of environmental friendliness. This is accomplished by lowering the amount of energy, chemicals, and waste produced.

### Applicability evaluation (BAGI tool, blueness evaluation)

One quantitative tool used to evaluate a technique’s feasibility in the field of analytical chemistry is BAGI^14^. The scores that are given vary from 25 to 100. A better level of practicality for the evaluated technique is indicated by a higher BAGI assessment score. This tool makes it easier to quickly determine a technique’s advantages and disadvantages in terms of applicability and allows for a comparative assessment of the efficacy of alternative analytical approaches.

## Conclusion

This study introduced a novel, eco-conscious RRS-enhancement method for the quantification of CAP, leveraging an ion-pairing strategy with erythrosin B in an aqueous medium. Beyond its analytical performance, the method was critically evaluated using multiple green chemistry metrics, confirming its environmental compatibility and practical viability. Importantly, the method’s simplicity—eliminating organic solvents and complex extraction steps—positions it as a sustainable alternative to conventional techniques. The high scores across ESA, AGREE, AGREE-prep, and NEMI frameworks underscore its alignment with green analytical chemistry principles. Moreover, the RGB 12 and BAGI assessments provided a multidimensional evaluation, revealing not only the method’s environmental friendliness but also its operational practicality. Although this study focused on tablet analysis, the method’s high sensitivity (LOD 5.61 ng mL⁻¹; LOQ 17 ng mL⁻¹) falls within the reported plasma levels of capivasertib, indicating its potential suitability for biological matrices. The CAP–EB ion-pair interaction is primarily electrostatic and is expected to remain stable in plasma, as supported by similar applications of xanthene dyes. The method is also compatible with simple, green sample-preparation steps such as protein precipitation. While plasma validation was not included here, the analytical performance suggests that the method can be readily extended to human plasma in future work. In summary, this work contributes a robust, green, and user-friendly analytical approach that aligns with modern sustainability goals, offering a valuable tool for both research and quality control laboratories.

## Data Availability

All data generated or analyzed during this study are included in this published article.
